# Dietary monounsaturated fatty acids reduce nerve inflammation and improve nerve function in murine models of obesity

**DOI:** 10.3389/fimmu.2026.1769812

**Published:** 2026-04-15

**Authors:** Nicolette V. Scott, Shubhi Yadav, Nafeesa A. Mahmood, Amy E. Rumora

**Affiliations:** 1 Department of Neurology, Columbia University Medical Center, New York, NY, United States; 2 Center for Motor Neuron Biology and Diseases, Columbia University Medical Center, New York, NY, United States; 3 Naomi Berrie Diabetes Center, Columbia University Medical Center, New York, NY, United States

**Keywords:** chemokine, cytokine, fatty acids, inflammation, neuropathy, nutraceutical, prediabetes

## Abstract

**Introduction:**

Peripheral neuropathy (PN) is a morbid and disabling condition that frequently develops during diabetes and prediabetes. In patients with type 2 diabetes and prediabetes, obesity is a major risk factor for PN indicating that dietary fatty acids may contribute to the molecular pathogenesis of PN. Recent evidence shows that overconsumption of dietary saturated fatty acids (SFAs) contributes to PN progression in prediabetic murine models of PN whereas monounsaturated fatty acids (MUFAs) improve nerve function without improving metabolic function, but the molecular basis for this differential impact on nerve function is incompletely understood. Fatty acids are known regulators of systemic inflammation, but the impact of SFAs and MUFAs on inflammation of the peripheral nervous system is not fully characterized.

**Methods:**

Herein, we used a mouse model of diet-induced obesity and prediabetic PN to determine if dietary SFAs and MUFAs alter nerve chemokines and cytokines, and to identify specific chemokines and cytokines that correlate with PN progression.

**Results:**

Mice fed an HFD enriched in SFAs (HFD-SFA) developed metabolic dysfunction and PN, as indicated by impaired hind paw withdrawal, decreased nerve conduction velocity (NCV), and loss of intraepidermal nerve fiber density (IENFD). Conversely, mice fed an HFD enriched in MUFAs (HFD-MUFA) for the duration of the study retained normal nerve function despite the development of metabolic dysfunction, and mice switched from HFD-SFA to HFD-MUFA regained nerve function. We next assessed the effect of dietary SFAs and MUFAs on peripheral nerve chemokines and cytokines using a multiplexing analysis to determine inflammatory factors underlying PN progression. Interestingly, a correlation analysis between PN phenotypes and chemokines and cytokines revealed a unique inflammatory profile associated with each PN phenotyping test. Impaired NCV in HFD-SFA mice correlated with elevated levels of sciatic nerve cytokines TNF-α and M-CSF, which was prevented or normalized by HFD-MUFA feeding. Impaired hind paw withdrawal in HFD-SFA mice correlated with changes in GM-CSF, IL-6, IL-9, IL-15, KC, MIP-1α, and VEGF.

**Discussion:**

These results indicate that dietary SFAs and MUFAs differentially impact chemokine and cytokine levels in the peripheral nerves of murine models of prediabetic PN, which may contribute to PN pathogenesis.

## Introduction

Diabetes is a major global health concern that impacts 537 million individuals worldwide, and another 352 million individuals have a condition that precedes diabetes; prediabetes (1). As the rate of diabetes and prediabetes diagnoses increase, there is a concomitant increase in comorbid complications (2, 3). Peripheral neuropathy (PN) is a prevalent neurological complication that develops in approximately half of diabetic patients and one third of prediabetic patients (4–6). Diabetic and prediabetic PN results in distal-to-proximal pain early in the course of the complication and eventually progresses to a loss of sensory function in the limbs. The pathophysiology of diabetic and prediabetic PN is characterized by a loss of distal sensory nerve fibers that progressively decline. Therapeutic treatments for diabetic PN are currently limited to glucose management, which has limited efficacy in individuals with type 2 diabetes (T2D) or prediabetes. Mounting evidence suggests that obesity and dietary fatty acids play an important role in PN progression (7–16), but the fatty acid-regulated molecular mechanisms underlying nerve damage are incompletely understood.

Preclinical murine models of prediabetes develop the same metabolic and neuropathic symptoms of PN as their human counterparts (14, 17–21). Both genetic T2D models (*db/db*) and high-fat diet (HFD) prediabetes models develop metabolic dysfunction including higher body weight, increased body fat mass, elevated fasting blood glucose, and impaired glucose tolerance (18, 22–25). Both models also develop PN characterized by decreased hind paw withdrawal responses, lower nerve conduction velocity (NCV), and decreased intraepidermal nerve fiber density (IENFD). These PN phenotypes correlate with the onset of dyslipidemia, and are reversed by dietary restriction (17), exercise (17, 26, 27), or dietary reversal to a low fat control diet (20, 23), indicating that restoring normolipidemia corrects early nerve damage associated with dyslipidemia in prediabetes. Interestingly, dietary intervention with an HFD enriched in MUFAs (HFD-MUFA) shows significant improvements in nerve function despite persistent metabolic dysfunction (28). Similarly, dietary supplementation with unsaturated fatty acids via Menhaden oil also improved nerve function in both streptozotocin-treated and HFD-fed murine models of diabetic and prediabetic neuropathy (29–31). These studies indicate that dietary fatty acids play an important role in the progression of prediabetic PN (32).

Inflammation of the peripheral nervous system has emerged as a possible contributor to PN progression in diabetic and prediabetic PN (3). One study evaluated changes in the inflammatory profile of sciatic nerve and dorsal root ganglion (DRG) during the course of PN development and progression in the *db/db* T2D mouse model, which develops robust PN by 16 weeks of age (22). PN was associated with significant alterations in interleukin-10 and interferon gamma as well as genes related to TREM1 signaling and granulocyte adhesion in the sciatic nerve from *db/db* mice (22). Toll-like receptor 4 (TLR4) also contributes to small fiber PN in streptozotocin-induced diabetic mouse models, and knockout of TLR4 prevents IENFD loss and macrophage accumulation (33). Another recent study showed that infiltration of C-C chemokine receptor type 2 (CCR2^+^) macrophages in the sciatic nerve preceded loss of peripheral nerve function in mice fed a high-fructose HFD (34). Ablating the recruitment of these CCR2^+^ macrophages in mice fed the high-fructose HFD caused a more rapid and severe degeneration of nerve fibers than in wildtype mice, indicating that CCR2+ macrophages delay nerve dysfunction and loss of IENFD (34). These studies indicate that inflammation contributes to the progression of PN in murine models of prediabetes and T2D. However, the effect of dietary fatty acids on nerve inflammatory factors during PN is incompletely understood.

We sought to determine whether dietary SFAs and MUFAs differentially impact nerve inflammation in murine models of prediabetic PN. We postulated that a HFD enriched with SFAs (HFD-SFA) would lead to accumulation of pro-inflammatory chemokines and cytokines within the peripheral nerves, whereas a HFD-MUFA would prevent nerve inflammation and improve nerve function in murine models of prediabetes. To test this, we conducted a multiplex analysis to quantitate the level of nerve and serum cytokines and chemokines in response to HFD-SFA and HFD-MUFA feeding. Interestingly, specific pro-inflammatory cytokines were elevated in the sciatic nerves of HFD-SFA mice, which correlated with impaired nerve function, whereas mice fed HFD-MUFA had reduced levels of pro-inflammatory cytokines. These findings suggest that the degree of saturation of dietary fatty acids has a distinct effect on nerve inflammation, which may provide new therapeutic targets for prediabetic PN.

## Materials and methods

### Mouse model

Four-week-old C57BL6/J mice were purchased from Jackson Laboratories (Catalog # 00664) and acclimated for one week. At 5 weeks of age, the mice were divided into four groups with 10 mice per group including (1) mice fed a standard diet (SD) (D12450J: 10% kcal fat, Research Diets) for the duration of the study from 5–30 weeks of age (2), mice fed an HFD-SFA (D12492: 60% kcal fat derived from lard, Research Diets) from 5–30 weeks of age (3), mice fed an HFD-MUFA (D18043009: 60% kcal fat derived from high MUFA sunflower oil, Research Diets) from 5–30 weeks of age and (4) a dietary intervention group with mice fed the HFD-SFA from 5–18 weeks of age followed by HFD-MUFA from 18–30 weeks of age (MUFA-DI). The fatty acid composition of the HFDs used in this study were reported previously (28). All mice were housed in a pathogen-free environment at the Columbia University Irving Medical Center Institute of Comparative Medicine, and all protocols adhered to the Columbia University Institutional Animal Care and Use Committee (Protocol number AC-AABL5564).

### Metabolic phenotyping

Metabolic phenotyping included monthly body weight measurements, bimonthly fasting blood glucose (FBG) and glucose tolerance test (GTT) measurements, and terminal body composition assessment. *FBG:* Mice were fasted for 4–6 hours prior to measuring FBG using an Alpha-Trak glucometer (Abbott Laboratories) (20, 21, 28). *GTTs:* GTTs were conducted by fasting mice for 4–6 hours, recording FBG for each mouse, followed by intraperitoneal injection of 1 g/kg of glucose. Blood glucose levels were measured with an Alpha-Trak glucometer at 15, 30, 60, and 120 minutes after glucose injection as described previously (20, 21, 28). *Body composition:* Fat and lean mass were measured for each mouse at 30 weeks using Echo-MRI-100H (EchoMRI LLC, Houston, TX) at the Columbia University Irving Medical Center (CUIMC) Russ Berrie Diabetes Research Center.

### Neuropathy phenotyping

Nerve function was evaluated in all mice at 18 weeks and 28–30 weeks of age using von Frey hind paw withdrawal behavioral phenotyping, NCV measurements, and IENFD quantitation.

#### von Frey hind paw withdrawal

All mice were subjected to von Frey hind paw withdrawal testing at both 18-weeks and 28-weeks. The mice were randomized and the tester was blinded to the identity of the mouse. All tests were conducted in a noise-proof and dimly lit room at the Mouse Neurobehavior core in the CUIMC Institute of Comparative Medicine to minimize external stimuli. Prior to testing, mice were acclimated for 2 hours on a metal mesh stand with each mouse in a separate compartment of a clear plexiglass enclosure. Following acclimation, a set of Semmes-Weinstein monofilaments (Stoelting^©^, Illinois) with a bending force range of 0.07 gram (g), 0.16 g, 0.4 g, 0.6 g, and 1.0 g were applied to the plantar surface of the hind paw in order from lowest force (0.07 g) to highest force (1.0g) and the response to the each filament was recorded by the tester. A positive response included a full withdrawal, a flinch, or toe-spread. The withdrawal response was measured 5 times for each hind paw totaling 10 measurements per mouse for each filament, and the test was repeated a total of 2–3 times. The first round of testing was considered part of the acclimation and was not included in the data analysis.

#### NCVs

Sural and sciatic NCVs were evaluated at both 18 weeks and 29 weeks as described previously (17, 20, 28). Briefly, mice were anesthetized with isoflurane and maintained at 1% isoflurane for the duration of the experiment. Sural NCV was measured by placing needle electrodes at the ankle and dorsum of the foot. The nerve was antidromically and supermaximally stimulated at the ankle and recorded at the dorsum of the foot. The sural NCV was determined by dividing the distance between the needle electrodes by the sensory nerve action potential latency. Sciatic-tibial NCVs were measured by stimulating orthodromically and supermaximally at the ankle and recording at the dorsum of the foot, followed by stimulation at the sciatic notch and recording at the dorsum of the foot. The distance between the ankle and dorsum of the foot was subtracted from the distance measured between the sciatic notch and the dorsum of the foot. This was then divided by the difference in ankle and notch latencies. All NCVs were conducted in accordance with guidelines by the Diabetic Complications Consortium (www.diacomp.org).

#### IENFD quantitation

The footpads were dissected from the plantar surface of the hind paw, fixed in Newcomer 2% Zamboni’s fixative for 4–6 hours followed by cryoprotection in 30% sucrose and embedding in OCT blocks. Footpads were sectioned to 30 μm sections and stained as previously described with PGP9.5 antibody (Proteintech, Catalog#14730-1-AP) primary antibody followed by a GFP-labeled secondary antibody as previously reported (35). A fluorescent z-stack of images was obtained on an SP8 Leica confocal microscope with an optical thickness of 3.3 μM using a 20X objective. Intraepidermal nerve fibers crossing the basement membrane to the epidermis were counted by a blinded tester using MetaMorph v 7.10.2 (Molecular devices) and Fiji v2.9.0 plugin NeuronJ image analysis software.

### Tissue collection and preparation

Sciatic nerve was pulverized with a pestle on dry ice and resuspended with cold PBS, pH 7.5. A modified Lowry protein assay was conducted on all samples to determine the total protein concentration per nerve sample. All samples were diluted to 2.25 mg/ml in PBS, pH 7.5, to normalize the total protein concentration across samples.

### Serum collection

To evaluate whether circulating chemokines and cytokines correlate with PN, blood samples were collected from the mouse tail vein at 18 and 30 weeks. Whole blood was collected into a sterile tube and incubated at room temperature for 30 minutes to promote clotting. The samples were then centrifuged at 1000 x g for 10 min at 4°C. Serum was collected into a pyrogen/endotoxin-free polypropylene tube, and diluted 2-fold into PBS, pH 7.5, as recommended by Eve Technologies^©^.

### Chemokine and cytokine analysis

Chemokine and cytokine levels were measured in 30-week sciatic nerve samples, 18-week serum samples, and 30-week serum samples. A Luminex xMAP technology was used for multiplexed quantification of 32 Mouse cytokines, chemokines, and growth factors including Eotaxin, granulocyte colony-stimulating factor (G-CSF), granulocyte-macrophage colony stimulating factor (GM-CSF), Interferon γ (IFNγ), Interleukin-1α (IL-1α), Interleukin-1β (IL-1β), Interleukin-2 (IL-2), Interleukin-3 (IL-3), Interleukin-4 (IL-4), Interleukin-5 (IL-5), Interleukin-6 (IL-6), Interleukin-7 (IL-7), Interleukin-9 (IL-9), Interleukin-10 (IL-10), Interleukin-12(p40) (IL-12(p40)), Interleukin-12(p70) (IL-12(p70)), Interleukin-13 (IL-13), Interleukin 15 (IL-15), Interleukin-17 (IL-17), inflammatory protein 10 (IP-10), Keratinocyte chemoattractant (KC), Leukemia inhibitory factor (LIF), Lipopolysaccharide-induced CXC chemokine (LIX), macrophage chemotactic protein-1 (MCP-1), macrophage colony stimulating factor (M-CSF), Monokine Induced by gamma Interferon (MIG), macrophage inflammatory protein-1α (MIP-1α), macrophage inflammatory protein-1β (MIP-1β), macrophage inflammatory protein-2 (MIP-2), Regulated upon Activation Normal T cell Expressed and Secreted (RANTES), Tumor Necrosis Factor α (TNFα), and vascular endothelial growth factor (VEGF). The 32-plex multiplexing analysis was conducted on a Luminex™ 200 system (Luminex, Austin, TX, USA) by Eve Technologies^©^ (Calgary, Alberta, Canada). The thirty-two markers were simultaneously measured in each sample with Eve Technologies’ Mouse Cytokine 32-Plex Discovery Assay^®^ (MilliporeSigma, Burlington, Massachusetts, USA) according to the manufacturer’s protocol. The concentration of each chemokine and cytokine was calculated based on a standard curve (**Supplementary Table 1**). Assay sensitivities of these markers ranged from 0.3 – 30.6 pg/mL for the 32-plex. Any chemokines or cytokines that were outside of the range of the standard curve were excluded.

### Statistical analysis

Metabolic and neuropathy measurements were analyzed by Ordinary one-way ANOVA with Tukey’s multiple comparison or an unpaired *t-*test. The association between chemokines and cytokines with PN phenotypes was evaluated using a two-tailed Pearson’s correlation analysis with a 95% confidence interval. Serum chemokine and cytokine association with PN phenotypes was evaluated using a two-tailed Pearson’s correlation analysis with a 95% confidence interval, and the concentration of each chemokine and cytokine at 18-weeks and 30-weeks was assessed with a Repeated Measure Two-way ANOVA followed by Fisher LSD test, HFD-SFA versus HFD-SFA to HFD-MUFA: #P<0.05.

## Results

### DIO mice develop prediabetes regardless of dietary fatty acid composition

**Figure 1:**
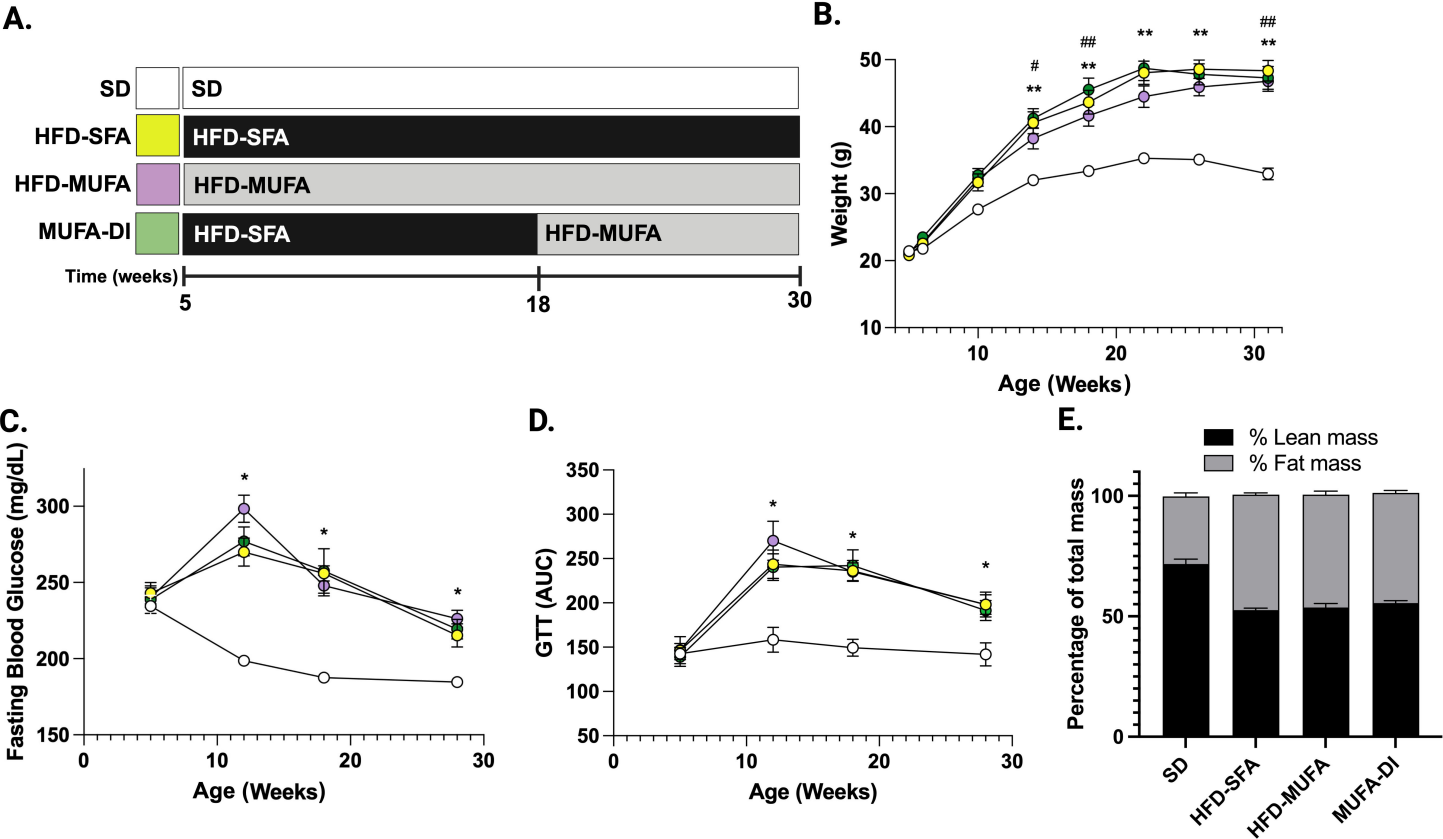
**(A)** The dietary paradigm consisted of four groups of mice fed 1) a low-fat standard diet (SD), 2) a high-fat diet (HFD) enriched in saturated fatty acids (SFAs, HFD-SFA), 3) a high-fat diet (HFD) enriched in monounsaturated fatty acids (MUFAs, HFD-MUFA), and 4) a dietary intervention group switched from HFD-SFA to HFD-MUFA (MUFA-DI) at 18 weeks. Metabolic measurements including **(B)** body weight (BW), **(C)** fasting blood glucose (FBG), and **(D)** glucose tolerance tests (GTTs) were evaluated monthly. **(E)** Body composition measurements were evaluated at the 30 week study termination. Serum samples were also collected and processed at 18 and 28–30 weeks to assess circulating changes in chemokines and cytokines. Ordinary one-way ANOVA with Tukey’s multiple comparisons; **p≤ 0.01, *p≤ 0.05, mice fed HFD-SFA versus SD; ##*p*≤ 0.01, #*p*≤ 0.05, mice fed HFD-MUFA versus SD.

Mice fed an HFD enriched in SFAs develop diet-induced obesity characterized by weight gain and metabolic dysfunction similar to T2D, prediabetes, and obese patients (20, 21, 23). Herein, we evaluated the effect of a HFD-SFA, HFD-MUFA, and MUFA-DI on both metabolic and nerve function longitudinally (**Figure 1A**). The HFD-SFA, HFD-MUFA, MUFA-DI mice gained weight throughout the duration of the study and were significantly heavier than SD mice by 14 weeks of age (**Figure 1B**). All HFD mice also had an elevated FBG and impaired glucose tolerance relative to SD mice starting at 12 weeks (**Figures 1C, D**). In line with longitudinal metabolic measures, all HFD mice also had a ~20% higher percent body fat mass than SD mice at study termination (**Figure 1E**). These results show that a HFD triggers metabolic dysfunction regardless of fatty acid composition similar to our previous findings (28) with metabolic dysfunction developing around 12 weeks of age.

### Dietary SFAs and MUFAs differentially regulate nerve function in DIO mice

We previously showed that PN developed by 16 weeks of age in mice fed a HFD-SFA (20, 21, 23), and switching HFD-SFA mice with PN to an HFD-MUFA for 8 weeks restored nerve function despite persistent metabolic dysfunction (28). Identifying the beneficial molecular effect of HFD-MUFA on nerve function during metabolic dysfunction could help to identify therapeutic targets for treating PN. Since dietary fatty acids differentially regulate circulating inflammatory factors (36–38), we sought to determine whether SFAs and MUFAs differentially regulate nerve-specific inflammation. We employed a similar dietary paradigm and measured sciatic nerve chemokines and cytokines that correlated with PN across the different dietary groups.

**Figure 2:**
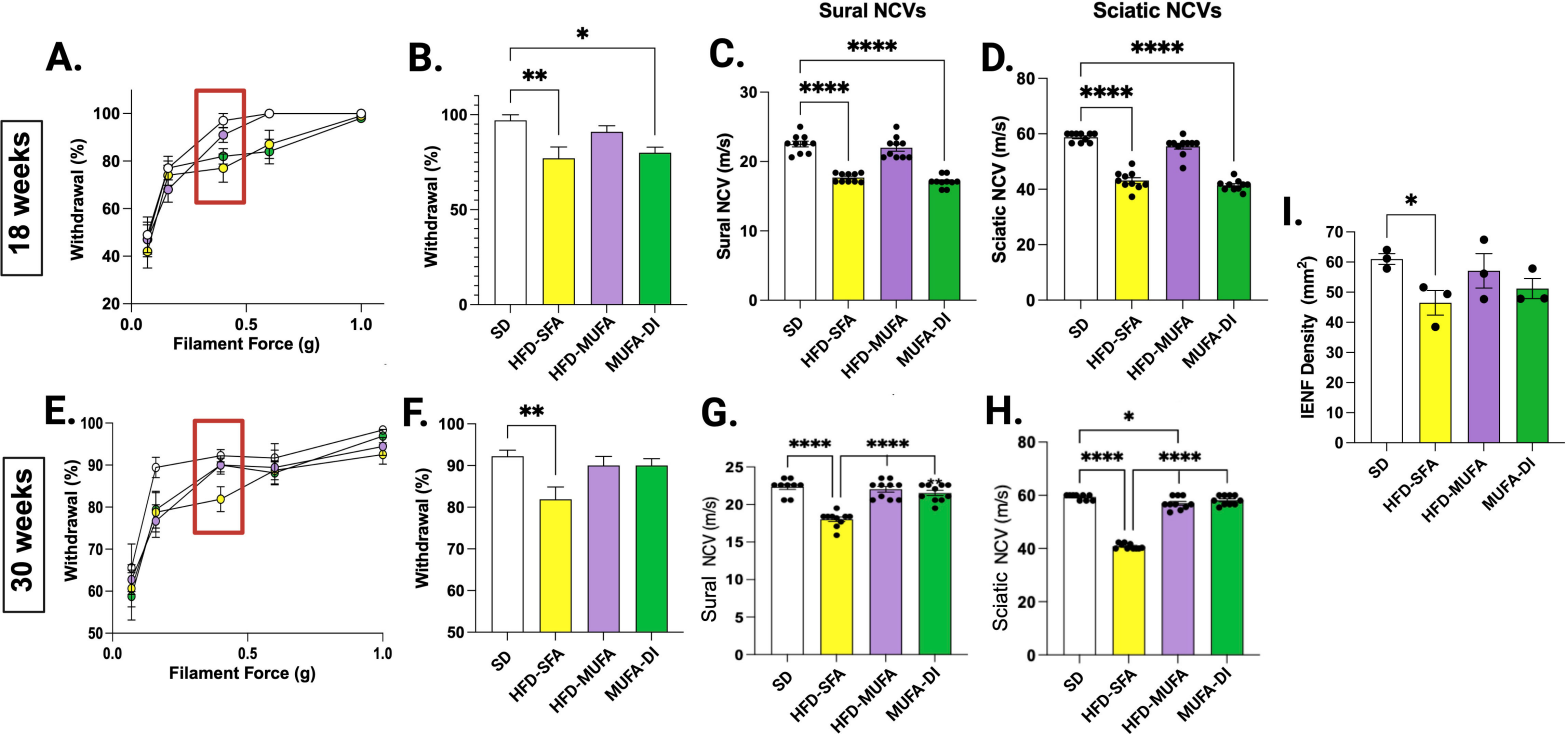
All mice were assessed for peripheral neuropathy (PN) phenotypes using behavioral, electrophysiological, and pathophysiological measurements. Hind paw withdrawal tests were conducted at 18 weeks **(A)** and 30 weeks **(E)** and responses were measured across von Frey filament forces (0.07 g, 0.16 g, 0.4 g, 0.6 g, and 1.0 g). The 0.4 g filament elicited the greatest difference between the four dietary groups and is quantitated at 18 weeks **(B)** and 30 weeks **(F)**. Nerve conduction velocity (NCV) of the **(C)** sural nerve and **(D)** sciatic nerve were measured at 18 weeks before the MUFA-DI and at **(G, H)** 30 weeks of age after the MUFA-DI. Finally, terminal intraepidermal nerve fiber densities (IENFDs) were quantitated at 30 weeks **(I)**. Ordinary one-way ANOVA with Tukey’s multiple comparisons; ****p≤ 0.0001, **p≤ 0.01, *p≤ 0.05.

We assessed the impact of dietary SFAs and MUFAs on PN phenotypes by measuring hind paw mechanical sensitivity, NCV, and IENFD count. Mice on the HFD-SFA showed a significant decrease in hind paw withdrawal compared to both the SD and HFD-MUFA groups at 18 weeks, indicating loss of mechanical sensitivity in HFD-SFA mice (**Figures 2A, B**). The most distinct change in hind paw withdrawal in HFD-SFA mice was visible with the 0.4 gram von Frey filament resulting in a significant ~15-20% loss of sensory function compared to SD mice and HFD-MUFA mice (**Figures 2A, B, E, F**). Similarly, HFD-SFA mice had a ~20 m/s lower sciatic NCV and ~6 m/s lower sural NCV (**Figures 2C, D, G, H**), indicating that sural and sciatic nerve function is diminished by HFD-SFA. To assess the pathophysiological impact of these diets, we measured footpad IENFD (**Figure 2I**), and found a significant loss of nerve fibers in foot pads from HFD-SFA mice compared to SD mice, indicative of axonal degeneration. Conversely, IENFD counts in foot pads from HFD-MUFA mice were not significantly different from SD mice.

MUFA-DI mice had impaired nerve function while on the HFD-SFA until 18 weeks, but benefitted from significant improvements in nerve function after 10 weeks on the HFD-MUFA (**Figure 2**). MUFA-DI mice showed a ~20% increase in mechanical sensitivity, 18 m/s increase in sciatic NCV, 6 m/s increase in sural NCV, and improved IENFD. These results indicate that a 10-week dietary intervention with MUFAs significantly improved PN phenotypes despite persistent metabolic dysfunction.

### Sciatic nerve TNFα and M-CSF levels correlate with nerve conduction changes

We next conducted a correlation analysis between individual chemokine/cytokine levels in the sciatic nerve and PN phenotypes to determine if specific chemokines or cytokines correlate with PN. Of the 32 chemokines and cytokines measured in sciatic nerve samples two were associated with NCVs and seven were associated with hind paw withdrawal responses (**Table 1**). Additionally, four serum chemokines and cytokines correlated with NCV and one correlated with hind paw withdrawal at either 18 weeks or 30 weeks (**Table 2**). This finding suggests that there may be specific chemokine or cytokine-driven neuroinflammatory changes that contribute to PN.

**Table 1:** Correlation analysis of nerve chemokines and cytokines with PN phenotypes.

chemokine/ cytokine	Sural nerve conduction velocity		Sciatic nerve conduction velocity		von Frey hind paw withdrawal	
	*p-value*	*r-value*	*p-value*	*r-value*	*p-value*	*r-value*
TNFα	0.0122	-0.3978	0.0160	-0.3833	:_	:_
M-CSF	0.0309	-0.3602	:_	:_	:_	:_
GM-CSF	:_	:_	:_	:_	0.0132	0.4038
IL-6	:_	:_	:_	:_	0.0488	0.3177
IL-9	:_	:_	:_	:_	0.0055	0.4364
IL-15	:_	:_	:_	:_	0.0071	0.4245
KC	:_	:_	:_	:_	0.0194	0.3729
MIP-1α	:_	:_	:_	:_	0.0118	0.4270
VEGF	:_	:_	:_	:_	0.0081	0.4179

**Table 2:** Correlation analysis of serum chemokines and cytokines with PN.

Time of serum collection	chemokine/ cytokine	Sural nerve conduction velocity		Sciatic nerve conduction velocity		von Frey hind paw withdrawal	
		*p-value*	*r-value*	*p-value*	*r-value*	*p-value*	*r-value*
18 weeks	LIX	0.0434	0.3252	0.0165	0.3816	:_	:_
	G-CSF	:_	:_	0.0174	0.3788	:_	:_
28–30 weeks	LIX	:_	:_	0.0396	0.3310	:_	:_
	IL-1β	:_	:_	0.0230	-0.3680	:_	:_
	IL-5	:_	:_	0.0263	-0.3651	:_	:_
	MCP-1	:_	:_	:_	:_	0.0083	-0.4222

**Figure 3:**
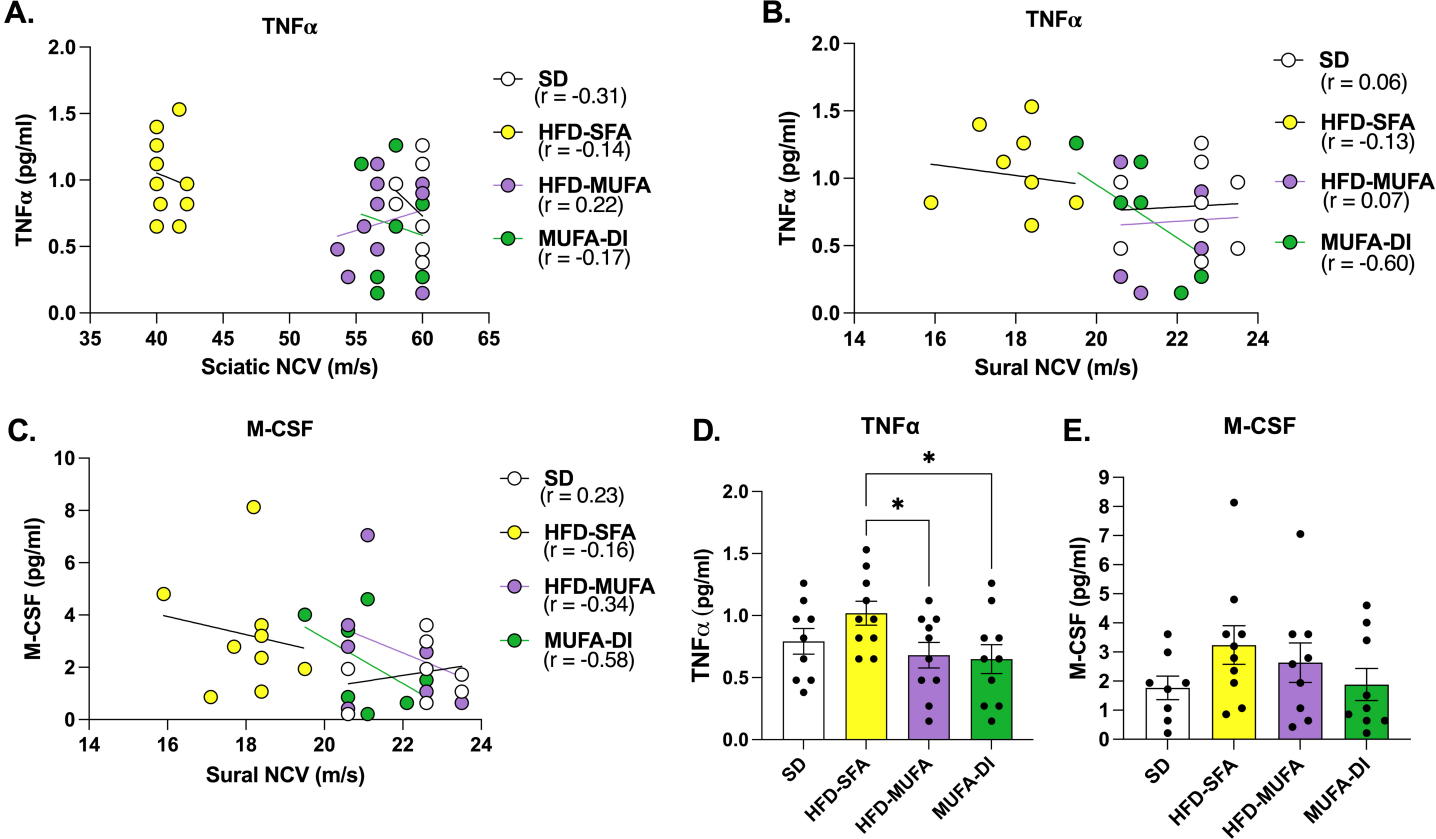
Correlations between sciatic nerve chemokines/cytokines and impaired NCV. **(A)** Sciatic nerve NCVs inversely correlated with the level of TNFα in the sciatic nerve at 30 weeks. **(B, C)** Sural nerve NCVs inversely correlated with the level of sciatic nerve TNFα and M-CSF at 30 weeks. **(D, E)** The TNFα and M-CSF level in sciatic nerve from mice fed HFD-SFA compared to SD, HFD-MUFA, and MUFA-DI at 30 weeks. Pearson’s two-tailed correlation analysis; Ordinary one-way ANOVA with Tukey’s multiple comparisons; *p≤ 0.05.

There was a significant inverse correlation between sciatic nerve TNFα levels and both sensory-motor and sensory NCVs at 28 weeks of age with a correlation coefficient of around -0.4 (**Figures 3A, B**), indicating a moderate negative correlation between TNFα level and NCVs. Sensory NCV was also inversely correlated with M-CSF (**Figure 3C**), a marker of macrophage proliferation and differentiation. Plotting the level of TNFα and M-CSF in sciatic nerve across the dietary groups showed that HFD-SFA induced an increase in both TNFα and M-CSF, whereas they were both lower in HFD-MUFA or MUFA-DI mice (**Figures 3D, E**). These results suggest that resident or infiltrating cells in the sciatic nerve produce TNFα and M-CSF in response to HFD-SFA and may contribute to diminished sciatic and sural nerve conduction in prediabetic PN.

### Sciatic nerve chemokine and cytokine levels correlate with hind paw withdrawal phenotypes

**Figure 4:**
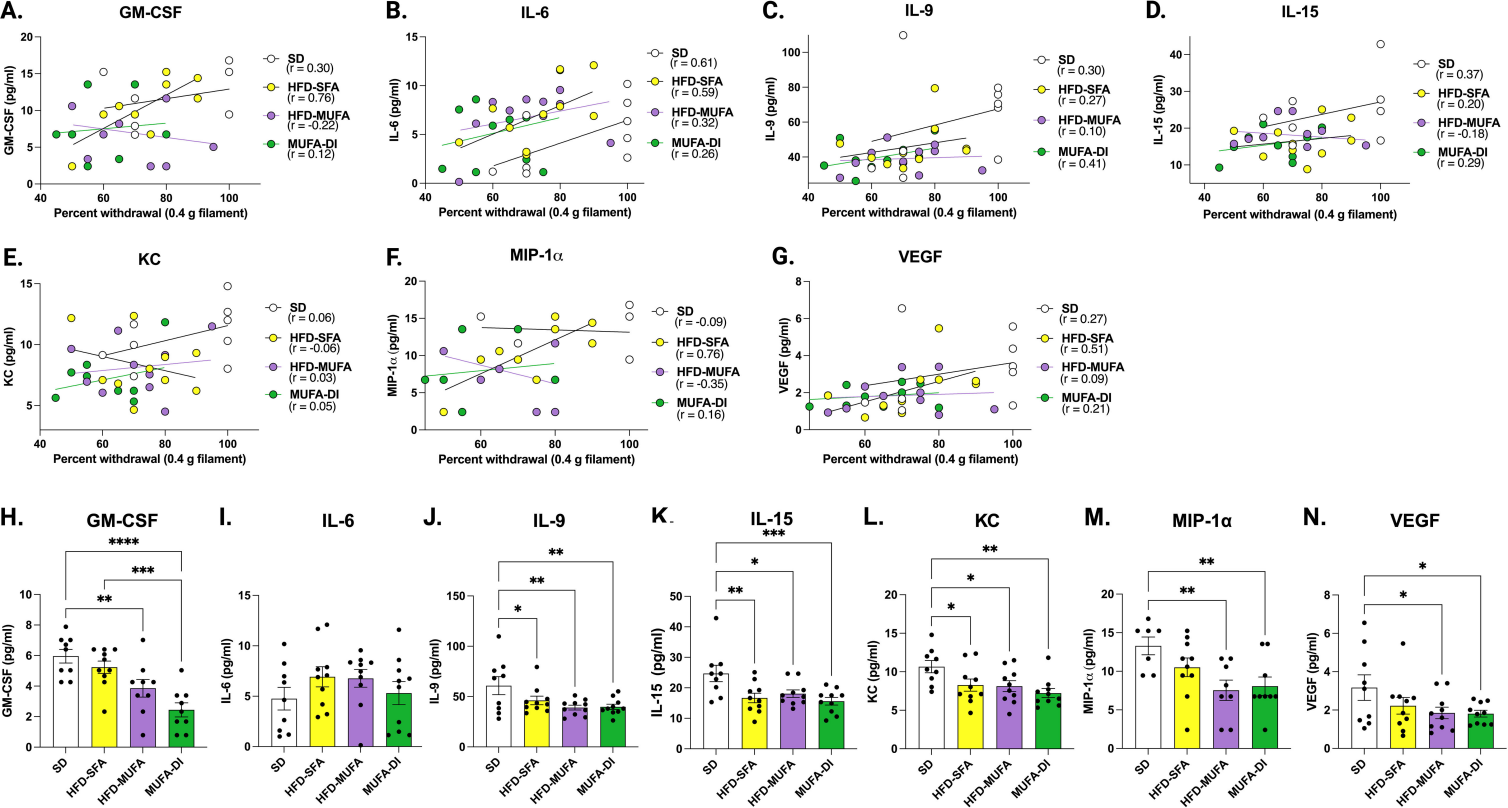
Correlations between sciatic nerve chemokines/cytokines and hind paw withdrawal. **(A–G)** Seven chemokines correlated with hind paw withdrawal at 30 weeks including **(A)** GM-CSF, **(B)** IL-6, **(C)** IL-9, **(D)** IL-15, **(E)** KC, **(F)** MIP-1α, and **(G)** VEGF. **(H–N)** The concentration of **(H)** GM-CSF, **(I)** IL-6, **(J)** IL-9, **(K)** IL-15, **(L)** KC, **(M)** MIP-1α, and **(N)** VEGF in pg/ml across all mouse groups at 30 weeks. Pearson’s two-tailed correlation analysis; Ordinary one-way ANOVA with Tukey’s multiple comparisons; ****p≤ 0.0001, ***p≤ 0.001, **p≤ 0.01, *p≤ 0.05.

A second correlation analysis was used to assess the association between sciatic nerve chemokine and cytokine levels and von Frey hind paw withdrawal phenotypes. Since the 0.4 g filament elicited significantly different withdrawal responses across dietary groups (**Figure 4**), the correlation was conducted using data obtained from this filament. This analysis identified seven chemokines and cytokines that correlated with changes in hind paw withdrawal across the dietary groups including GM-CSF, IL-6, IL-9, IL-15, KC, MIP-1α, and VEGF (**Figures 4A–G****).** Plots of the correlations showed that GM-CSF, IL-6, MIP-1α, VEGF resulted in strong positive correlation coefficient values in mice fed HFD-SFA, indicating that high levels of these chemokines in the sciatic nerve are associated with better hind paw withdrawal and are indicative of a higher response rate to the 0.4 g filament stimulus (**Figures 4A, B, F, G**). Conversely, correlation plots for HFD-MUFA and MUFA-DI showed low correlation coefficient values for GM-CSF, IL-6, MIP-1α, VEGF, suggesting these chemokines and cytokines are not associated with hind paw withdrawal in non-neuropathic HFD-MUFA-fed mice and MUFA-DI mice with improved nerve function. All seven sciatic nerve chemokines and cytokines except IL-6 were significantly decreased in mice fed HFD-MUFA or with MUFA-DI, indicating that lower chemokine and cytokine levels in these mice is associated with nerve function (**Figures 4H–N**). Sciatic nerve IL-9, IL-15, and KC had low levels in HFD-SFA, HFD-MUFA and MUFA-DI mice compared to the SD mice, implying that these chemokines and cytokines are impacted by a HFD regardless of the type of fatty acid. Interestingly, the chemokines and cytokines associated with hind paw withdrawal were different from those associated with nerve conduction changes in **Figure 3**.

### Specific serum chemokine and cytokine levels correlate with nerve conduction changes

**Figure 5:**
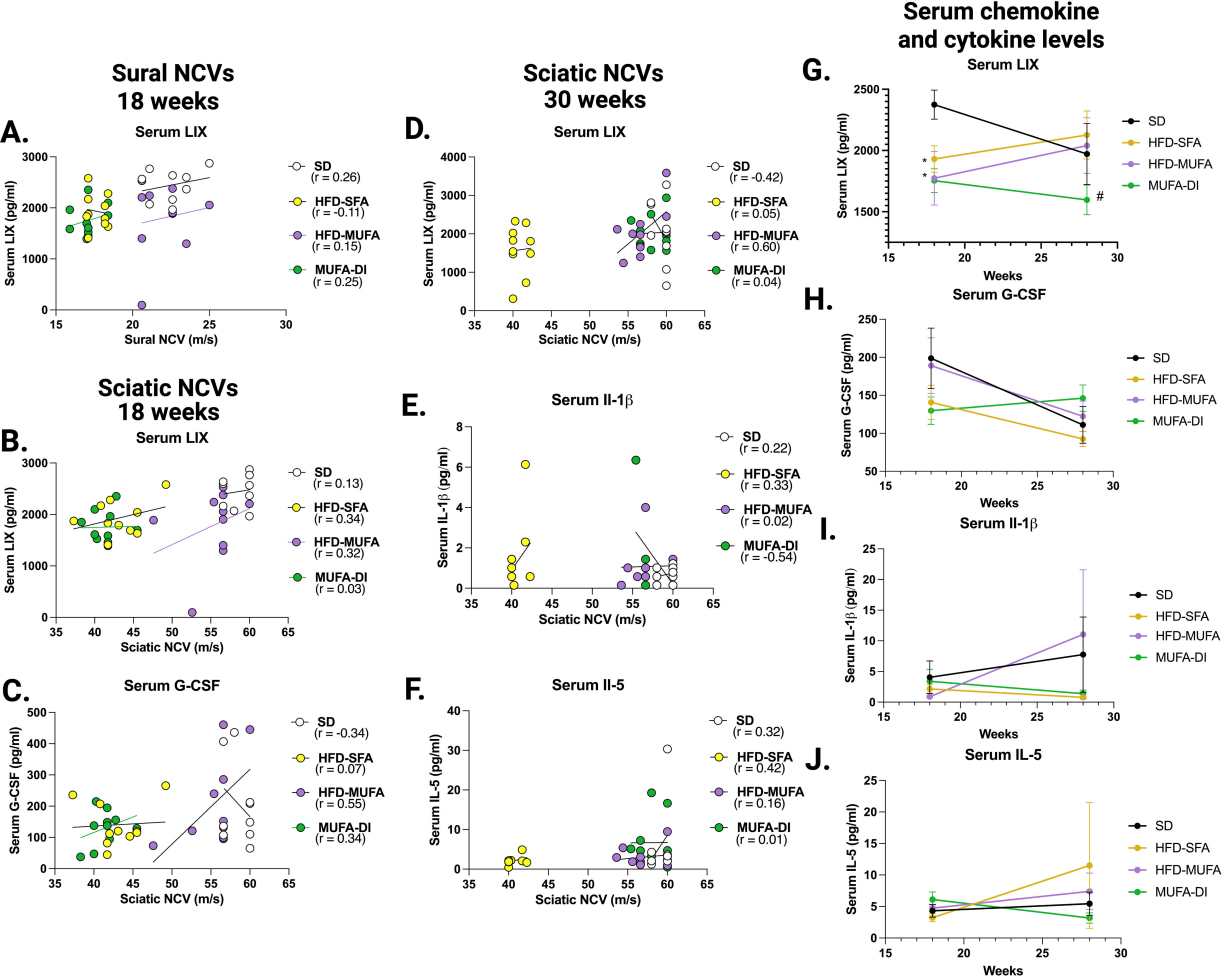
Serum chemokine and cytokine levels associated with alterations in NCV at the 18-week midpoint and 30-week termination of the study. **(A)** Only the serum cytokine LIX correlated with sural nerve function at 18 weeks. At 18 weeks serum **(B)** LIX and **(C)** G-CSF correlated with sciatic NCV. At 30 weeks serum **(D)** LIX, **(E)** IL-1β, and **(F)** IL-5 correlated with sciatic NCV. The concentration of serum **(G)** LIX, **(H)** G-CSF, **(I)** IL-1β, and **(J)** IL-5 was compared between 18 and 30 weeks. Pearson’s two-tailed correlation analysis; Ordinary one-way ANOVA with Tukey’s multiple comparisons; *p≤ 0.05, mice fed HFD-MUFA or MUFA-DI versus SD; #*p*≤ 0.05, mice fed HFD-SFA versus MUFA-DI.

**Figure 6:**
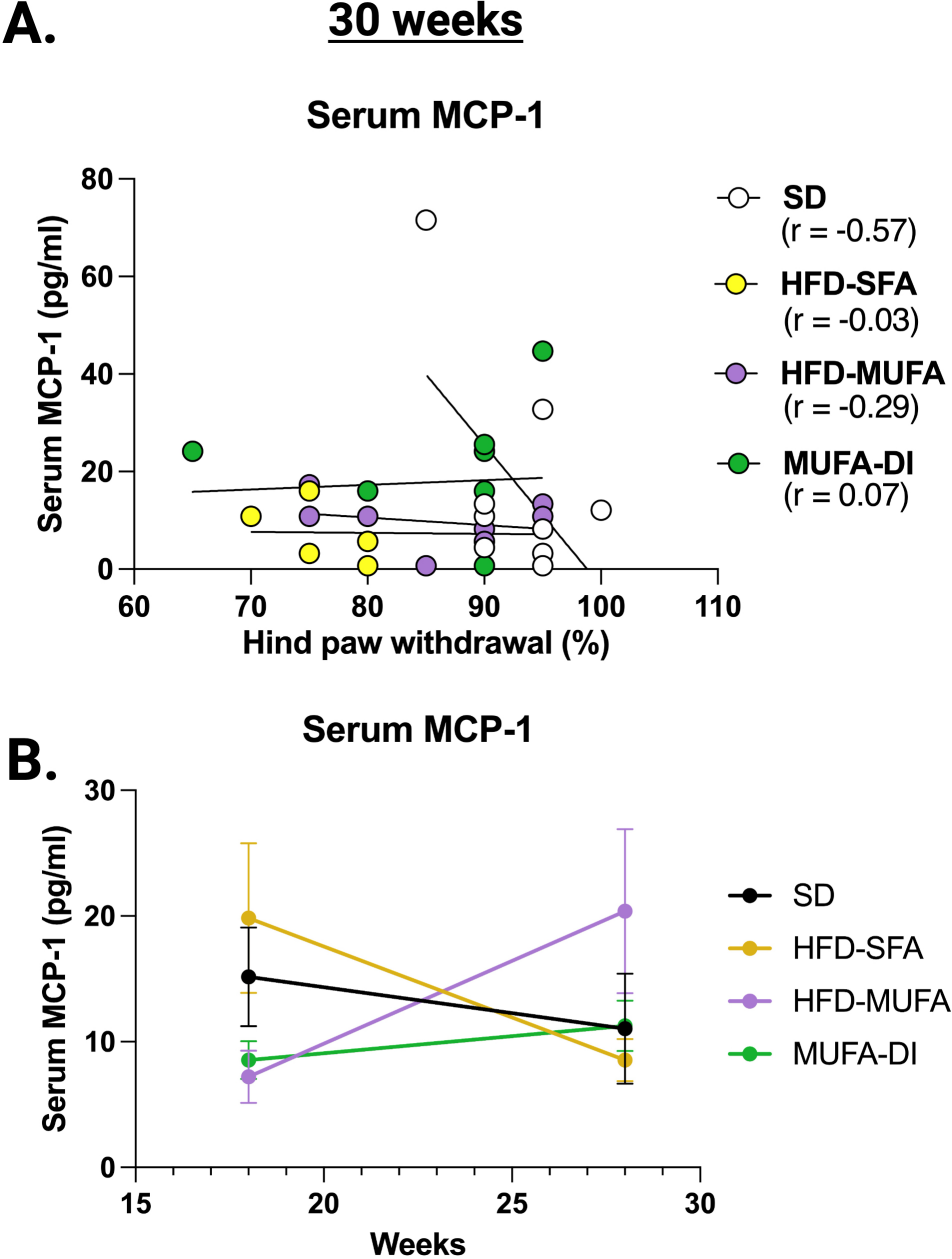
Serum chemokine/cytokine levels associated with alterations in von Frey hind paw withdrawal. At the 30-week termination of the study, **(A)** serum MCP-1 was the sole circulating chemokine correlating with hind paw withdrawal phenotypes and **(B)** serum MCP-1 level was altered from 18 to 30 weeks of age. Pearson’s two-tailed correlation analysis; Ordinary one-way ANOVA with Tukey’s multiple comparisons.

To determine whether specific circulating chemokines or cytokines parallel PN progression in HFD mice, changes in serum chemokines and cytokines were measured at the 18-week midpoint and the 30-week termination of the study. LIX was the only cytokine that correlated with sural NCV at 18 weeks (**Figure 5A**). LIX also showed a positive correlation with sciatic NCV at both 18-weeks and 30-weeks (**Figures 5A, B, D**), indicating that circulating LIX level may correlate with nerve function during prediabetes and obesity. The levels of IL-1β, IL-5, and LIX show significant changes in concentration at the mid-point and termination of the study (**Figure 5**). IL-1β and IL-5 were inversely correlated with PN at 28–30 weeks whereas LIX was positively associated with sciatic NCV at 28–30 weeks (**Table 2**). Additionally, MCP-1 levels were correlated with hind paw withdrawal at the 28–30 week time point and increased in concentration from 18 weeks to 30 weeks with HFD-MUFA and MUFA-DI compared to the HFD-SFA, which decreased from 18 to 30 weeks of age (**Table 2**, **Figure 6**).

## Discussion

PN develops in 30-50% of patients with diabetes and prediabetes marked by progressive loss of sensory function, for which there is currently no treatment. PN in pre-clinical models is associated with transcriptional and cellular inflammatory remodeling within the peripheral nervous system, but the specific inflammatory factors associated with changes in nerve function and the dietary factors that regulate them are less understood. Herein, we used a pre-clinical murine model of prediabetes to test the effect of a HFD-SFA and HFD-MUFA on nerve chemokine and cytokine levels. The HFD-SFA impaired nerve function, while HFD-MUFA or MUFA-DI showed normal nerve function despite equivalent metabolic dysfunction, similar to our previous study (28), indicating that dietary fatty acids differentially regulate nerve function. Multiplex analysis of chemokines and cytokines in the sciatic nerve showed that impaired NCV was associated with increased TNFα and M-CSF levels. Interestingly, a different set of chemokines and cytokines including GM-CSF, IL-6, IL-9, IL-15, KC, MIP-1α, and VEGF were associated with impaired hind paw withdrawal. These studies indicate that specific chemokines and cytokines are associated with PN progression, potentially providing distinct and testable therapeutic targets.

We evaluated the effect of dietary fatty acids on nerve inflammatory chemokines and cytokines in an established mouse model of PN and prediabetes. C57BL6 mice are widely used to study PN associated with obesity and prediabetes because they develop robust PN associated with metabolic dysfunction, including higher body weight, elevated fasting blood glucose, impaired glucose tolerance, and increased body fat mass, analogous to their human counterparts (7, 10, 11, 39). Herein, mice were fed a 60% HFD enriched in either SFAs or MUFAs, and a third group was fed an SFA-rich HFD followed by a dietary intervention with a MUFA-rich HFD. All HFD groups developed metabolic dysfunction regardless of fatty acid composition of the diet, but only mice fed a HFD-SFA developed PN. Interestingly, mice fed a MUFA-rich HFD did not develop PN, and mice with MUFA-DI benefitted from a significant restoration in nerve function. These results support our previous findings that SFAs and MUFAs differentially regulate nerve function (28), as well as findings from other studies showing the beneficial effect of unsaturated fatty acid-rich oils for slowing the progression of PN in pre-clinical models of prediabetes (29, 31, 40–42). Our HFD feeding paradigm started at 5 weeks of age resulting in PN at both 18 and 30 weeks of age in HFD-SFA mice, but not HFD-MUFA and MUFA-DI mice. A separate study evaluated the efficacy of HFD feeding rats from 12–28 weeks of age followed by supplementation with dietary oils of varying fatty acid composition for an additional 32 weeks (29). Interestingly, the authors found that oils rich in omega-3 polyunsaturated fatty acids significantly improved peripheral nerve function, whereas other oils, including MUFA-rich oils, resulted in a partial rescue of nerve function. This indicates that the beneficial impact of dietary fatty acids for PN may depend on the time of exposure to the fatty acids, but this possibility warrants further investigation. Similar studies in human subjects including the National Health and Nutrition Examination Survey study showed lower incident neuropathy associated with dietary PUFA intake in diabetic subjects (43), while other studies identified significant improvements in T2D PN in response to linolenic acid (44, 45) and eicosapentaenoic acid (46). Despite a clear association between dietary unsaturated fatty acids and improvements in diabetic PN, the molecular basis for the differential effect of SFAs and MUFAs on nerve function is not completely understood.

To determine whether dietary SFAs and MUFAs differentially effect nerve inflammation, we quantitated the level of sciatic nerve chemokines and cytokines associated with alterations in nerve function. Elevated sciatic nerve TNFα in HFD-SFA mice correlated with impaired NCV at both 18 and 30 weeks, whereas sciatic nerve TNFα level was significantly lower in HFD-MUFA mice with normal nerve function and MUFA-DI mice with improved nerve function. These results indicate that dietary SFAs may trigger increases in TNFα-mediated nerve damage during PN, and that dietary MUFAs minimize TNFα-mediated nerve damage in the context of prediabetes and obesity. TNFα is a pro-inflammatory cytokine that becomes dysregulated by obesity-related metabolic diseases (47), modulates cellular lipid metabolism (48), and may be a key contributing factor to the pro-inflammatory effects of HFD-SFA during PN. TNFα is a major regulator of PN in type 1 diabetes, and inhibition of TNFα with a TNFα inhibitor infliximab reduced PN in Type 1 diabetic PN (49), but follow up studies showed that infliximab had off-target anti-inflammatory effects that improved nerve function in T1D mice. Although the association between TNFα and peripheral neuropathies has been previously described, the regulation of sciatic nerve TNFα by dietary fatty acids is novel and may be a feasible therapeutic target for obesity-driven PN.

Interestingly, M-CSF was also inversely correlated with sural NCV, but not sciatic NCV, despite the trending increase in M-CSF expression in the sciatic nerve. This result indicates that HFD-SFA triggers elevated levels of M-CSF in the sciatic nerve that are detrimental for sensory nerve NCV. Since the sciatic nerve is composed of both sensory and motor nerve fibers, M-CSF may be produced specifically by injured sensory nerve fibers in the sciatic nerve thereby affecting only sensory NCV. M-CSF is crucial for the recruitment, polarization, and expansion of monocytes and macrophages during neuropathic pain (50, 51). Although M-CSF is most commonly produced by monocytes, macrophages, fibroblasts, endothelial cells, and lymphocytes (51), DRG sensory neurons have also been reported to amplify M-CSF production and secretion in response to nerve injury to recruit macrophages to the site of injury (52). However, accumulation of macrophages within the nerves leads to neuropathic pain sensitization (52). The HFD mouse model used herein develops neuropathic pain early between 8–12 weeks of age (26), followed by a loss of sensory function by 16 weeks of age (20, 23, 28). Therefore, the inverse correlation between M-CSF and sensory NCVs suggests that HFD-SFA damages sensory neurons and M-CSF upregulation could be a compensatory mechanism to recruit macrophages for sensory nerve fiber repair and improved DRG sensitization. In support of this conjecture, M-CSF stimulates macrophage M2 polarization which stimulates the production of anti-inflammatory cytokines and chemokines (50). A separate study evaluated the impact of a HFD-SFA on sciatic nerve macrophages and found elevated levels of sciatic nerve F4/80+ macrophages correlating with painful PN at 12 weeks on the HFD prior to axonal degeneration (34). These findings indicate that protective F4/80+ macrophages may accumulate in the sciatic nerve at 12 weeks during painful PN, but persistent intake of SFAs until 24–30 weeks may lead to loss of F4/80+ macrophages in the sciatic nerve corresponding with reduced sensory function and axonal loss in HFD-SFA PN. Assessment of inflammatory changes that contribute to sensory deficits throughout the course of PN will be essential to understand the inflammatory cascade that underlies PN progression during metabolic dysfunction.

Hind paw withdrawal behavioral phenotypes also correlated with changes in sciatic nerve cytokines and chemokines including GM-CSF, IL-6, IL-9, IL-15, KC, MIP-1α, and VEGF, and were unique from those that correlated with NCVs. Of these, GM-CSF, IL-6, MIP-1α, and VEGF were strongly correlated with hind paw withdrawal phenotypes, indicating a possible compensatory mechanism in the sciatic nerve in response to distal HFD-SFA-induced nerve damage in the plantar surface of the hind paw. These chemokines and cytokines are reported to modulate macrophage recruitment, maturation, and polarization (53). GM-CSF is considered a potential target for the treatment of inflammation due to its robust role triggering macrophages to polarize to the M1 state and produce pro-inflammatory cytokines including IL-6 (51), which also correlated with changes in hind paw withdrawal. MIP-1α is a chemokine secreted by macrophages to recruit and activate monocytes, macrophages, and neutrophils at a site of injury (53). Lastly, there is a strong link between VEGF and macrophage recruitment to support angiogenesis (54, 55). Altogether our data suggests that macrophage-related chemokines and cytokines (53) are likely to be differentially regulated by SFAs and MUFAs. These findings parallel the report that macrophages slow the progression of PN in the sciatic nerve of HFD fed mice (34).

Several serum chemokines and cytokines correlated with alterations in nerve function and may be circulating markers of impaired nerve conduction and sensory dysfunction. LIX was associated with changes in NCV, while MCP-1 parallel changes in hind paw withdrawal behaviors. Interestingly, there was no overlap between serum and sciatic nerve chemokines and cytokines that correlated with PN phenotypes. This result suggests that fatty acids trigger inflammatory changes within the nerve that are unique from systemic inflammatory changes during HFD feeding. Another possibility is that circulating chemokines and cytokines may act on sensory neurons thereby triggering specific inflammatory cascades within the nerve. Serum LIX (also known as CXCL5 in humans) correlated with NCV in our study. Interestingly, CXCL5 also acts directly on sensory neurons triggering pain and downstream inflammatory cascades in a model of arthritis (56), suggesting that CXCL5 may trigger nerve inflammation. Similarly, IL-1β is reported to correlate with diabetic neuropathy and stimulate hypersensitivity in sensory neurons (57). Altogether, the lack of overlap between serum and sciatic nerve chemokine and cytokine changes herein is indicative of a tissue-specific sciatic nerve inflammatory response.

We also found that specific chemokines/cytokines correlated with different PN measures. These results may indicate that the inflammatory profile changes as PN progresses distally to proximally. TNFα and M-CSF were inversely correlated with NCV indicating that the inflammatory signature associated with PN in each nerve type may vary as PN progresses. Future studies will focus on defining these chemokine/cytokine changes as PN progresses, and determine whether these inflammatory signatures are indicative of immune cell type recruitment.

To better understand the pathophysiology of prediabetic PN, it will be important to identify the cell type that produces TNFα in the sciatic and sural nerves. TNFα can be produced by a number of different resident and infiltrating cell types in the peripheral nervous system, but the cell type(s) that produce TNFα throughout the progression of PN during prediabetes and obesity are unknown. One possibility is that resident cells within the peripheral nervous system produce TNFα as a signal to recruit immune cells to the nerve. Schwann cells, the primary glial cell of the peripheral nervous system, produce TNFα in response to pathogenic stimuli including nerve compression injury (58) and LPS stimulation (59). Schwann cells sheath the axons of sensory neurons providing support for fast saltatory conduction, metabolic support, and axonal health. Alternatively, higher TNFα may result from cellular inflammatory signaling pathways. TLR-4 signaling was recently discovered to play a major role in the progression of small fiber sensory PN in diabetic mice, and deletion of TLR4 prevented sensory PN (33). TLR-4 signaling causes high expression of pro-inflammatory TNFα and IL-6 (60), which may contribute to alterations in TNFα level.

Our study had some limitations. First, the study was conducted only in male mice. Although previous reports show no sex dimorphic differences in PN in HFD murine models (61), there are often sex-specific differences in chemokine and cytokine profiles that can contribute to inflammatory differences (62–64). Future studies will compare the impact of dietary SFAs and MUFAs on nerve chemokine and cytokine changes in both male and female mice. Second, chemokine and cytokine measurements were only evaluated in the sciatic nerve and could not be evaluated in the sensory DRG or sural nerves using a multiplex analysis due to the limited tissue size of the DRG and sural nerves. Third, although alterations in nerve chemokines and cytokines correlated with PN phenotypes, the mechanistic connection between inflammatory factors and PN progression is incompletely understood. Future endeavors will focus on identifying the cell type within the sciatic nerve that produces elevated levels of TNFα during HFD-SFA feeding. The level and localization of these chemokines and cytokines will also be evaluated in the distal sural nerve and proximal DRG using immunohistochemistry. Fourth, we did not evaluate the impact of vascular dysfunction on inflammation and axonal degeneration in HFD-SFA, HFD-MUFA, and MUFA-DI mice. It will be interesting to explore the possible connection between inflammation and microvascular dysfunction during PN in HFD-SFA mice, and to assess whether HFD-MUFA prevents microvascular damage. Finally, blocking or lowering nerve TNFα and M-CSF in mice fed HFD-SFA or supplementing these cytokines during HFD-MUFA feeding will define the functional role of these inflammatory factors in PN.

Our study suggests that dietary SFAs and MUFAs differentially regulate inflammatory chemokines and cytokines during PN and prediabetes. One particularly interesting finding is the difference in chemokines and cytokines that correlate PN phenotypes including impaired NCV (proximal) and hind paw withdrawal (distal) indicating a possible distal to proximal gradient in inflammatory signaling. Additionally, we identified several serum chemokines and cytokines that were differentially regulated by SFAs and MUFAs and are potential circulating markers of PN in obesity and prediabetes. Finally, these findings emphasize the importance of dietary factors in regulating peripheral nervous system inflammation and PN pathogenesis, and suggest that dietary MUFAs are a potential nutraceutical for diabetic and prediabetic PN.

## Data Availability

The original contributions presented in the study are included in the article/**Supplementary Material**. Further inquiries can be directed to the corresponding author.

## References

[1] International Diabetes Federation . IDF diabetes atlas. 11th edn. Brussels, Belgium: International Diabetes Federation (2025).

[2] Eid SA Rumora AE Beirowski B Bennett DL Hur J Savelieff MG . New perspectives in diabetic neuropathy. Neuron. (2023) 111:2623–41. doi: 10.1016/j.neuron.2023.05.003. PMID: 37263266 PMC10525009

[3] Stino AM Rumora AE Kim B Feldman EL . Evolving concepts on the role of dyslipidemia, bioenergetics, and inflammation in the pathogenesis and treatment of diabetic peripheral neuropathy. J Peripher Nerv Syst. (2020) 25:76–84. doi: 10.1111/jns.12387. PMID: 32412144 PMC7375363

[4] Gordois A Scuffham P Shearer A Oglesby A Tobian JA . The health care costs of diabetic peripheral neuropathy in the US. Diabetes Care. (2003) 26:1790–5. doi: 10.2337/diacare.26.6.1790. PMID: 12766111

[5] Pop-Busui R Boulton AJ Feldman EL Bril V Freeman R Malik RA . Diabetic neuropathy: A position statement by the American Diabetes Association. Diabetes Care. (2017) 40:136–54. doi: 10.2337/dc16-2042. PMID: 27999003 PMC6977405

[6] Callaghan BC Price RS Feldman EL . Distal symmetric polyneuropathy: A review. JAMA. (2015) 314:2172–81. doi: 10.1136/jnnp-2014-307575. PMID: 26599185 PMC5125083

[7] Callaghan BC Gao L Li Y Zhou X Reynolds E Banerjee M . Diabetes and obesity are the main metabolic drivers of peripheral neuropathy. Ann Clin Transl Neurol. (2018) 5:397–405. doi: 10.1002/acn3.531. PMID: 29687018 PMC5899909

[8] Callaghan BC Hur J Feldman EL . Diabetic neuropathy: One disease or two? Curr Opin Neurol. (2012) 25:536–41. doi: 10.1097/WCO.0b013e328357a797, PMID: 22892951 PMC4239661

[9] Callaghan BC Reynolds E Banerjee M Chant E Villegas-Umana E Feldman EL . Central obesity is associated with neuropathy in the severely obese. Mayo Clin Proc. (2020) 95:1342–53. doi: 10.1016/j.mayocp.2020.03.025. PMID: 32622444 PMC7340115

[10] Callaghan BC Xia R Banerjee M de Rekeneire N Harris TB Newman AB . Metabolic syndrome components are associated with symptomatic polyneuropathy independent of glycemic status. Diabetes Care. (2016) 39:801–7. doi: 10.2337/dc16-0081. PMID: 26965720 PMC4839175

[11] Callaghan BC Xia R Reynolds E Banerjee M Rothberg AE Burant CF . Association between metabolic syndrome components and polyneuropathy in an obese population. JAMA Neurol. (2016) 73:1468–76. doi: 10.1001/jamaneurol.2016.3745. PMID: 27802497 PMC5217829

[12] Christensen DH Knudsen ST Gylfadottir SS Christensen LB Nielsen JS Beck-Nielsen H . Metabolic factors, lifestyle habits, and possible polyneuropathy in early type 2 diabetes: A nationwide study of 5,249 patients in the Danish Centre for Strategic Research in Type 2 Diabetes (DD2) cohort. Diabetes Care. (2020) 43:1266–75. doi: 10.2337/dc19-2277. PMID: 32295810

[13] Elafros MA Andersen H Bennett DL Savelieff MG Viswanathan V Callaghan BC . Towards prevention of diabetic peripheral neuropathy: Clinical presentation, pathogenesis, and new treatments. Lancet Neurol. (2022) 21:922–36. doi: 10.1016/s1474-4422(22)00188-0. PMID: 36115364 PMC10112836

[14] Guo K Savelieff MG Rumora AE Alakwaa FM Callaghan BC Hur J . Plasma metabolomics and lipidomics differentiate obese individuals by peripheral neuropathy status. J Clin Endocrinol Metab. (2022) 107:1091–109. doi: 10.1210/clinem/dgab844. PMID: 34878536 PMC8947234

[15] Rumora AE Guo K Alakwaa FM Andersen ST Reynolds EL Jorgensen ME . Plasma lipid metabolites associate with diabetic polyneuropathy in a cohort with type 2 diabetes. Ann Clin Transl Neurol. (2021) 8(6):1292–307. doi: 10.1002/acn3.51367. PMID: 33955722 PMC8164865

[16] Savelieff MG Callaghan BC Feldman EL . The emerging role of dyslipidemia in diabetic microvascular complications. Curr Opin Endocrinol Diabetes Obes. (2020) 27:115–23. doi: 10.1097/med.0000000000000533. PMID: 32073426 PMC11533224

[17] Eid SA Elzinga SE Kim B Rumora AE Hayes JM Carter A . High-intensity interval training, caloric restriction, or their combination have beneficial effects on metabolically acquired peripheral neuropathy. Diabetes. (2024) 73(11):1895–907. doi: 10.2337/figshare.26764066. PMID: 39163551 PMC11493763

[18] Guo K Figueroa-Romero C Noureldein M Hinder LM Sakowski SA Rumora AE . Gut microbiota in a mouse model of obesity and peripheral neuropathy associated with plasma and nerve lipidomics and nerve transcriptomics. Microbiome. (2023) 11:52. doi: 10.1186/s40168-022-01436-3. PMID: 36922895 PMC10015923

[19] Noureldein MH Rumora AE Teener SJ Rigan DM Hayes JM Mendelson FE . Dietary fatty acid composition alters gut microbiome in mice with obesity-induced peripheral neuropathy. Nutrients. (2025) 17(4):737. doi: 10.3390/nu17040737. PMID: 40005065 PMC11858455

[20] O’Brien PD Guo K Eid SA Rumora AE Hinder LM Hayes JM . Integrated lipidomic and transcriptomic analyses identify altered nerve triglycerides in mouse models of prediabetes and type 2 diabetes. Dis Model Mech. (2020) 13:1754–8411. doi: 10.1242/dmm.042101, PMID: 31822493 PMC6994925

[21] Rumora AE Guo K Hinder LM O’Brien PD Hayes JM Hur J . A high-fat diet disrupts nerve lipids and mitochondrial function in murine models of neuropathy. Front Physiol. (2022) 13:921942. doi: 10.3389/fphys.2022.921942. PMID: 36072849 PMC9441493

[22] Hinder LM Murdock BJ Park M Bender DE O’Brien PD Rumora AE . Transcriptional networks of progressive diabetic peripheral neuropathy in the db/db mouse model of type 2 diabetes: An inflammatory story. Exp Neurol. (2018) 305:33–43. doi: 10.1016/j.expneurol.2018.03.011. PMID: 29550371 PMC5955815

[23] Hinder LM O’Brien PD Hayes JM Backus C Solway AP Sims-Robinson C . Dietary reversal of neuropathy in a murine model of prediabetes and metabolic syndrome. Dis Model Mech. (2017) 10:717–25. doi: 10.1242/dmm.028530. PMID: 28381495 PMC5483005

[24] McGregor BA Eid S Rumora AE Murdock B Guo K de Anda-Jauregui G . Conserved transcriptional signatures in human and murine diabetic peripheral neuropathy. Sci Rep. (2018) 8:17678. doi: 10.1038/s41598-018-36098-5. PMID: 30518872 PMC6281650

[25] Sas KM Kayampilly P Byun J Nair V Hinder LM Hur J . Tissue-specific metabolic reprogramming drives nutrient flux in diabetic complications. JCI Insight. (2016) 1:e86976. doi: 10.1172/jci.insight.86976. PMID: 27699244 PMC5033761

[26] Groover AL Ryals JM Guilford BL Wilson NM Christianson JA Wright DE . Exercise-mediated improvements in painful neuropathy associated with prediabetes in mice. Pain. (2013) 154:2658–67. doi: 10.1016/j.pain.2013.07.052. PMID: 23932909 PMC3844098

[27] Cooper MA Kluding PM Wright DE . Emerging relationships between exercise, sensory nerves, and neuropathic pain. Front Neurosci. (2016) 10:372. doi: 10.3389/fnins.2016.00372. PMID: 27601974 PMC4993768

[28] Rumora AE LoGrasso G Hayes JM Mendelson FE Tabbey MA Haidar JA . The divergent roles of dietary saturated and monounsaturated fatty acids on nerve function in murine models of obesity. J Neurosci. (2019) 39:3770–81. doi: 10.1523/jneurosci.3173-18.2019. PMID: 30886017 PMC6510336

[29] Coppey L Davidson E Shevalye H Torres ME Yorek MA . Effect of dietary oils on peripheral neuropathy-related endpoints in dietary obese rats. Diabetes Metab Syndr Obes. (2018) 11:117–27. doi: 10.2147/dmso.s159071. PMID: 29674850 PMC5898889

[30] Coppey LJ Davidson EP Obrosov A Yorek MA . Enriching the diet with menhaden oil improves peripheral neuropathy in streptozotocin-induced type 1 diabetic rats. J Neurophysiol. (2015) 113:701–8. doi: 10.1152/jn.00718.2014. PMID: 25376787 PMC4312862

[31] Davidson EP Coppey LJ Shevalye H Obrosov A Yorek MA . Effect of dietary content of menhaden oil with or without salsalate on neuropathic endpoints in high-fat-fed/low-dose streptozotocin-treated Sprague Dawley rats. J Diabetes Res. (2018) 2018:2967127. doi: 10.1155/2018/2967127. PMID: 30057911 PMC6051246

[32] Rumora AE Kim B Feldman EL . A role for fatty acids in peripheral neuropathy associated with type 2 diabetes and prediabetes. Antioxid Redox Signal. (2022) 37:560–77. doi: 10.1089/ars.2021.0155. PMID: 35152728 PMC9499450

[33] Crowards S Ryals J Heslop L Hauser W Totta-Griese G Madden T . TLR4 deletion reduces small fiber sensory abnormalities and nerve degeneration in diabetic male mice. Neuroscience. (2025) 589:268–79. doi: 10.1016/j.neuroscience.2025.10.052. PMID: 41167527 PMC13112499

[34] Hakim S Jain A Adamson SS Petrova V Indajang J Kim HW . Macrophages protect against sensory axon loss in peripheral neuropathy. Nature. (2025) 640:212–20. doi: 10.1038/s41586-024-08535-1. PMID: 39939762 PMC11964918

[35] Cheng HT Dauch JR Hayes JM Yanik BM Feldman EL . Nerve growth factor/p38 signaling increases intraepidermal nerve fiber densities in painful neuropathy of type 2 diabetes. Neurobiol Dis. (2012) 45:280–7. doi: 10.1016/j.nbd.2011.08.011. PMID: 21872660 PMC3225563

[36] Hung HC Tsai SF Chou HW Tsai MJ Hsu PL Kuo YM . Dietary fatty acids differentially affect secretion of pro-inflammatory cytokines in human THP-1 monocytes. Sci Rep. (2023) 13:5511. doi: 10.1038/s41598-023-32710-5. PMID: 37016048 PMC10073224

[37] Fritsche KL . The science of fatty acids and inflammation. Adv Nutr. (2015) 6:293S–301S. doi: 10.3945/an.114.006940. PMID: 25979502 PMC4424767

[38] Poli A Agostoni C Visioli F . Dietary fatty acids and inflammation: Focus on the n-6 series. Int J Mol Sci. (2023) 24(5):4567. doi: 10.3390/ijms24054567. PMID: 36901998 PMC10003459

[39] Andersen ST Witte DR Dalsgaard EM Andersen H Nawroth P Fleming T . Risk factors for incident diabetic polyneuropathy in a cohort with screen-detected type 2 diabetes followed for 13 years: ADDITION-Denmark. Diabetes Care. (2018) 41:1068–75. doi: 10.2337/dc17-2062. PMID: 29487078

[40] Coppey LJ Shevalye H Obrosov A Davidson EP Yorek MA . Determination of peripheral neuropathy in high-fat diet fed low-dose streptozotocin-treated female C57Bl/6J mice and Sprague-Dawley rats. J Diabetes Investig. (2018) 9:1033–40. doi: 10.1111/jdi.12814. PMID: 29412513 PMC6123046

[41] Davidson E Obrosov O Coppey L Yorek M . Omega-3 polyunsaturated fatty acids effect on neuropathy of the peripheral nervous system in dietary obese rats: Is the source of these fatty acids important for outcome? Diabetes Metab Syndr Obes. (2025) 18:2709–21. doi: 10.2147/dmso.s522285. PMID: 40799308 PMC12341837

[42] Davidson E Obrosov O Coppey L Yorek M . Omega-3 polyunsaturated fatty acids (PUFAs) and diabetic peripheral neuropathy: A pre-clinical study examining the effect of omega-3 PUFAs from fish oil, krill oil, algae or pharmaceutical-derived ethyl esters using type 2 diabetic rats. Biomedicines. (2025) 18:2709–721. doi: 10.3390/biomedicines13071607. PMID: 40722680 PMC12292556

[43] Tao M McDowell MA Saydah SH Eberhardt MS . Relationship of polyunsaturated fatty acid intake to peripheral neuropathy among adults with diabetes in the National Health and Nutrition Examination Survey (NHANES) 1999 2004. Diabetes Care. (2008) 31:93–5. doi: 10.2337/dc07-0931. PMID: 17914029

[44] Jamal GA Carmichael H . The effect of gamma-linolenic acid on human diabetic peripheral neuropathy: A double-blind placebo-controlled trial. Diabetes Med. (1990) 7:319–23. doi: 10.1111/j.1464-5491.1990.tb01397.x. PMID: 2159860

[45] Keen H Payan J Allawi J Walker J Jamal GA Weir AI . Treatment of diabetic neuropathy with gamma-linolenic acid. The gamma-linolenic acid multicenter trial group. Diabetes Care. (1993) 16:8–15. doi: 10.2337/diacare.16.1.8. PMID: 8380765

[46] Okuda Y Mizutani M Ogawa M Sone H Asano M Asakura Y . Long-term effects of eicosapentaenoic acid on diabetic peripheral neuropathy and serum lipids in patients with type II diabetes mellitus. J Diabetes Complications. (1996) 10:280–7. doi: 10.1016/1056-8727(95)00081-x. PMID: 8887017

[47] Wang Y Guo L Yin X McCarthy EC Cheng MI Hoang AT . Pathogenic TNF-alpha drives peripheral nerve inflammation in an Aire-deficient model of autoimmunity. Proc Natl Acad Sci USA. (2022) 119(4):e2114406119. doi: 10.1073/pnas.2114406119. PMID: 35058362 PMC8795502

[48] Chen X Xun K Chen L Wang Y . TNF-alpha, a potent lipid metabolism regulator. Cell Biochem Funct. (2009) 27:407–16. doi: 10.1002/cbf.1596. PMID: 19757404

[49] Yamakawa I Kojima H Terashima T Katagi M Oi J Urabe H . Inactivation of TNF-alpha ameliorates diabetic neuropathy in mice. Am J Physiol Endocrinol Metab. (2011) 301:E844–52. doi: 10.1152/ajpendo.00029.2011. PMID: 21810933 PMC3213998

[50] Hamilton TA Zhao C Pavicic PG Datta S . Myeloid colony-stimulating factors as regulators of macrophage polarization. Front Immunol. (2014) 5:554. doi: 10.3389/fimmu.2014.00554. PMID: 25484881 PMC4240161

[51] Lee KMC Achuthan AA Hamilton JA . GM-CSF: a promising target in inflammation and autoimmunity. Immunotargets Ther. (2020) 9:225–40. doi: 10.2147/ITT.S262566, PMID: 33150139 PMC7605919

[52] Grosu AV Gheorghe RO Filippi A Deftu AF Isler M Suter M . Dorsal root ganglia CSF1(+) neuronal subtypes have different impact on macrophages and microglia after spared nerve injury. J Peripher Nerv Syst. (2024) 29:514–27. doi: 10.1111/jns.12674. PMID: 39581686 PMC11625985

[53] Ousman SS David S . MIP-1alpha, MCP-1, GM-CSF, and TNF-alpha control the immune cell response that mediates rapid phagocytosis of myelin from the adult mouse spinal cord. J Neurosci. (2001) 21:4649–56. doi: 10.1523/jneurosci.21-13-04649.2001. PMID: 11425892 PMC6762369

[54] Sekiguchi K Ito Y Hattori K Inoue T Hosono K Honda M . VEGF receptor 1-expressing macrophages recruited from bone marrow enhances angiogenesis in endometrial tissues. Sci Rep. (2019) 9:7037. doi: 10.1038/s41598-019-43185-8. PMID: 31065021 PMC6504918

[55] Wheeler KC Jena MK Pradhan BS Nayak N Das S Hsu CD . VEGF may contribute to macrophage recruitment and M2 polarization in the decidua. PloS One. (2018) 13:e0191040. doi: 10.1371/journal.pone.0191040. PMID: 29324807 PMC5764356

[56] Yin C Liu B Dong Z Shi S Peng C Pan Y . CXCL5 activates CXCR2 in nociceptive sensory neurons to drive joint pain and inflammation in experimental gouty arthritis. Nat Commun. (2024) 15:3263. doi: 10.1038/s41467-024-47640-7. PMID: 38627393 PMC11021482

[57] Sher EK Prnjavorac B Farhat EK Palic B Ansar S Sher F . Effect of diabetic neuropathy on reparative ability and immune response system. Mol Biotechnol. (2023). doi: 10.1007/s12033-023-00813-z. PMID: 37523019 PMC13518443

[58] Wagner R Myers RR . Schwann cells produce tumor necrosis factor alpha: expression in injured and non-injured nerves. Neuroscience. (1996) 73:625–9. doi: 10.1016/0306-4522(96)00127-3. PMID: 8809782

[59] Cheng C Qin Y Shao X Wang H Gao Y Cheng M . Induction of TNF-alpha by LPS in Schwann cell is regulated by MAPK activation signals. Cell Mol Neurobiol. (2007) 27:909–21. doi: 10.1007/s10571-007-9215-4. PMID: 17902045 PMC11517400

[60] Sameer AS Nissar S . Toll-like receptors (TLRs): structure, functions, signaling, and role of their polymorphisms in colorectal cancer susceptibility. BioMed Res Int. (2021) 2021:1157023. doi: 10.1155/2021/1157023. PMID: 34552981 PMC8452412

[61] Elzinga SE Savelieff MG O’Brien PD Mendelson FE Hayes JM Feldman EL . Sex differences in insulin resistance, but not peripheral neuropathy, in a diet-induced prediabetes mouse model. Dis Model Mech. (2021) : 14(4):1754–8411. doi: 10.1242/dmm.048909. PMID: 33692086 PMC8077554

[62] Girdhar K Mine K DaCosta JM Atkinson MA Ludvigsson J Altindis E . Sex-specific cytokine, chemokine, and growth factor signatures in T1D patients and progressors. FASEB J. (2024) 38:e70270. doi: 10.1101/2024.09.05.611513. PMID: 39704278 PMC11660211

[63] Leung J Jayachandran M Kendall-Thomas J Behrenbeck T Araoz P Miller VM . Pilot study of sex differences in chemokine/cytokine markers of atherosclerosis in humans. Gend Med. (2008) 5:44–52. doi: 10.1016/s1550-8579(08)80007-1. PMID: 18420165

[64] Simoes E Correia-Lima J Sardas L Storti F Otani T Vasques DAC . Sex dimorphism in inflammatory response to obesity in childhood. Int J Obes (Lond). (2021) 45:879–87. doi: 10.1038/s41366-021-00753-1. PMID: 33526854 PMC8005372

